# *QuickStats*: Age-Adjusted Percentage* of Adults Aged ≥18 Years Who Had an Influenza Vaccination in the Past 12 Months,^†^ by Sex and Race/Ethnicity^§^ — National Health Interview Survey, United States, 2019^¶^

**DOI:** 10.15585/mmwr.mm7014a6

**Published:** 2021-04-09

**Figure:**
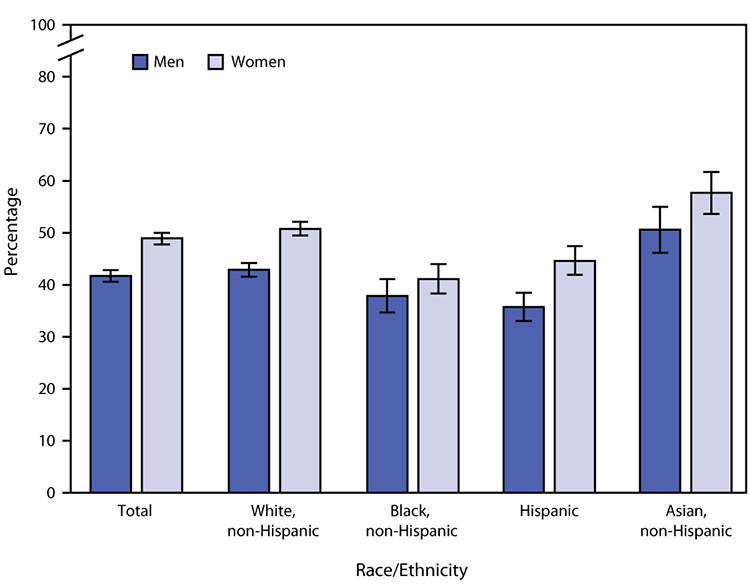
In 2019, women aged ≥18 years were more likely than were men (48.9% versus 41.7%) to have had an influenza vaccination in the past 12 months. This pattern was found for non-Hispanic White adults (50.8% versus 42.9%), Hispanic adults (44.6% versus 35.7%), and non-Hispanic Asian adults (57.7% versus 50.7%), but there was no statistically significant difference by sex among non-Hispanic Black adults (41.1% versus 37.9%). For both men and women, non-Hispanic Black and Hispanic adults were less likely to have had an influenza vaccination in the past 12 months than were non-Hispanic White and non-Hispanic Asian adults.

**Source:** National Center for Health Statistics, National Health Interview Survey, 2019. https://www.cdc.gov/nchs/nhis.htm

